# Modeling purple basil, sage, spearmint, and sweet basil responses to daily light integral and mean daily temperature

**DOI:** 10.1371/journal.pone.0294905

**Published:** 2023-11-30

**Authors:** Kellie J. Walters, Sean Tarr, Roberto G. Lopez

**Affiliations:** 1 Plant Sciences Department, University of Tennessee, Knoxville, TN, United States of America; 2 Department of Horticulture, Michigan State University, East Lansing, MI, United States of America; Institute for Biological Research, University of Belgrade, SERBIA

## Abstract

Mean daily temperature (MDT) and daily light integral (DLI) can interact to influence growth and development of plants. Our objectives were to determine 1) the extent DLI and MDT influence growth and development of purple basil ‘Dark Opal’ (*Ocimum basilicum*), sage ‘Extrakta’ (*Salvia officinalis*), spearmint ‘Spanish’ (*Mentha spicata*), and sweet basil ‘Nufar’ (*Ocimum basilicum*) and 2) the influence on purple basil color. Young plants were transplanted into hydroponic systems in five greenhouse compartments with MDT set points of 23, 26, 29, 32, or 35°C and DLIs from 5 to 19 mol·m^‒2^·d^‒1^, respectively. At harvest, growth, development, and leaf color was measured. Branch number of all genera increased as MDT increased from ~23 to 35°C. Sweet basil branch number increased as DLI increased from 5.5 to 13.2 mol·m^‒2^·d^‒1^, but the effect of DLI was attenuated as MDT decreased. In contrast, increasing DLI from ~5–6 to ~18–19 mol·m^‒2^·d^‒1^ increased sage and spearmint branch number more when MDT was lower (~23°C) compared to ~35°C, while branch number of purple basil was not influenced by DLI. The optimal MDT (MDT_opt_) for sage and spearmint fresh mass decreased from 27.5 to 23.5°C and from 30.4 to 27.8°C, respectively, as DLI increased from 6 to 18 mol·m^‒2^·d^‒1^, while sweet basil fresh mass MDT_opt_ increased from 32.6 to 35.5°C as DLI increased from 6 to 11 mol·m^‒2^·d^‒1^. Purple basil was greener [hue angle (h°) = 99° to 138°] when MDT was ~35°C regardless of DLI, but when MDT was lower (~25°C), basil was more purple (h° = 335°) at a DLI of 18.7 compared to 5.0 mol·m^‒2^·d^‒1^ (h° = 98°). Taken together, MDT and DLI can have a large impact on plant growth, development, and color and can be manipulated to achieve desired characteristics.

## Introduction

Culinary herbs are increasingly being produced in controlled environments (CE) due to their high value, relatively short production duration, limited post-harvest life, and compact height, making them conducive for both greenhouse and indoor vertical production. From 1998 to 2014, protected environment fresh cut culinary herb production in the United States increased in value by 58% (adjusted for inflation: $26 million increase) and 16.4 million kg were produced under 1.3 million m^2^ of protected production area [1,2]. CE agriculture (CEA), including greenhouse and indoor production, creates the opportunity to precisely control the environment to potentially increase crop productivity and quality, provide a consistent year-round supply of locally grown food, and increase food safety and security; additionally, recent technological advances have improved the economic feasibility of CEA [3–5]. Though the ability to precisely control the growing environment exists, so does a knowledge gap in how to use the technology to its greatest potential. If known, increased production efficiencies including greater yields, increased energy use efficiency, and higher quality products could be realized.

Both light (radiant energy) and temperature (thermal energy) play large roles in plant growth, development, and quality. While radiant energy is measured instantaneously, plants largely integrate the accumulation of radiant energy over the course of a 24-h period. Therefore, the daily light integral (DLI; mol·m^‒2^·d^‒1^) is a useful calculation to integrate fluctuations of radiant energy in a greenhouse to meaningful numbers to predict plant responses over time [6]. Photosynthesis, and thus biomass production, growth, and secondary metabolite production, are largely driven by radiation. Growing under low DLIs, such as in greenhouse production during the winter months, can result in low-quality and low-yielding crops [6]. Increasing the DLI from 9.3 to 17.8 mol·m^‒2^·d^‒1^ during indoor sweet basil ‘Improved Genovese Compact’ (*Ocimum basilicum*) production not only increased fresh mass 78%, but also anthocyanin concentrations [7]. Since increasing DLI can increase anthocyanin concentrations, a phenolic pigment contributing to blue, red, and purple coloration, we hypothesized that increasing DLI would not only improve biomass production, but also make purple basil (*Ocimum basilicum*) appear more purple and less green.

Plant developmental rates, including leaf unfolding rate and progress towards flowering, are primarily influenced by temperature [8–11]. However, temperature also plays a role in growth and yield. Similar to radiation, plant response to temperature is often integrated over one day and is referred to as the mean daily temperature (MDT). The relationship between MDT and plant growth and development can be described by the temperature response curve. Below the species- and cultivar-specific base temperature (T_b_), plant growth and development does not progress. As temperature increases above T_b_, plant growth and development increase linearly or near-linearly until reaching the optimum temperature (T_opt_), where the growth or developmental rate is the greatest. Increased enzymatic activity caused by increased temperature are largely responsible for the temperature-dependent increases in photosynthesis and growth in the linear range between T_b_ and T_opt_ [12]. As MDT increases above T_opt_, growth and development rates decrease until the maximum temperature (T_max_), beyond which plant development ceases.

Several researchers have investigated the influence of temperature on culinary herbs. Chang et al. [13] determined the T_b_ for sweet basil ‘Genovese’ growth was 10.9°C. Similarly, Walters and Currey [11] determined the T_b_ for growth of holy basil ‘holy’ (*Ocimum tenuiflorum*), sweet basil ‘Nufar’, lemon basil ‘Sweet Dani’, and lemon basil ‘Lime’ (*Ocimum* ×*citriodorum*), were 10.9, 11.3, 11.6, and 12.1°C, respectively. Chang et al. [13] found that growth of sweet basil ‘Genovese’ increased as temperature increased from 15 to 25°C, but plateaued as temperature further increased to 30°C. Therefore, the T_opt_ for sweet basil ‘Genovese’ was between 25 and 30°C when the DLI was 20 to 22 mol·m^‒2^·d^‒1^. Walters and Currey [11] estimated the T_opt_ of basil was between 29 and 35°C when the DLI was 19.5 mol·m^‒2^·d^‒1^. For basil, T_max_ is a very high temperature not commonly reached in CEA production. In another study, six species of sage besides *Salvia officinalis* (common sage) were grown for 36 d with 15-h daytime temperatures of 20, 25, 30, 35, or 40°C and 9-h nights of 15 or 20°C; maximum dry weight occurred at MDTs ranging from 24 to 37°C and varied among species [14]. For common sage, fresh mass increased from 37 to 72 g and plant height increased from 9 to 18 cm as temperature increased from 18 to 27°C [15]. Additionally, metabolic processes including pigment production are influenced by MDT; as temperature increases, anthocyanin concentrations decrease, leading to less red/blue/purple plants [16–19].

Temperature and radiation can interact to influence plant growth and development. Not only overt characteristics can be explained and modeled by a temperature response curve, including leaf unfolding rate and biomass accumulation, but also metabolic processes including photosynthesis and respiration increase as temperature increases to a process-specific T_opt_. While examining the interaction of radiation and temperature on photosynthesis, researchers have determined that T_opt_ generally increases as radiation intensity increases [8,20]. For example, the carbon dioxide (CO_2_) assimilation T_opt_ was 5 to 10°C when the radiation intensity was low (205 μmol·m^–2^·s^–1^) but increased to 27°C when the radiation intensity increased to 2,050 μmol·m^–2^·s^–1^ for non-light or -temperature acclimated carnation ‘Cerise Royalette’ (*Dianthus caryophyllus*) [20]. Similarly, the T_opt_ of both light-acclimated and non-acclimated cucumber ‘Moskovsky Teplichnyi’ (*Cucumis sativus*) seedlings increased as DLI increased from ~3 to ~13 mol·m^‒2^·d^‒1^ [8].

Models to predict plant growth and development in response to MDT and DLI have been generated for many floriculture crops including celosia ‘Gloria Mix’ (*Celosia argentea*) [21], cyclamen ‘Metis Scarlet Red’ (*Cyclamen persicum*) [22], impatiens ‘Accent Red’ (*Impatiens walleriana*) [21], marigold ‘Bonanza Yellow’ (*Tagetes patula*) [23], pansy ‘Universal Violet’ (*Viola* ×*wittrockiana*) [24,25], petunia ‘Easy Wave Coral Reef’ and ‘Wave Purple’ (*Petunia ×hybrida*) [26], and salvia ‘Vista Red’ (*Salvia splendens*) [23]. While models including single environmental parameters have been generated to predict growth and development of culinary herbs, to our knowledge, multiparameter models including MDT and DLI have not been published; these data are integral in increasing production efficiencies, yield, and quality in CEs.

Our objectives were to 1) determine the extent DLI and MDT influence the growth and development of purple basil, sage, spearmint (*Mentha spicata*), and sweet basil; and 2) determine to what extent DLI and MDT influence purple basil color. We hypothesized the optimal MDT (MDT_opt_) of each species would increase as DLI increases. We also hypothesized there would be an interactive effect of DLI and MDT on purple basil color, where increasing MDT will result in a less purple, more green plant and increasing the DLI will increase the purple color more when the MDT is lower but have no effect at higher MDTs.

## Materials and methods

### Plant production

Purple basil ‘Dark Opal’, common sage ‘Extrakta’, and sweet basil ‘Nufar’ seeds were sown in phenolic foam cubes (2.5 × 2.5 × 4 cm, Horticube XL; Smithers Oasis, Kent, OH) and the flats were placed in a greenhouse. Spearmint ‘Spanish’ vegetative stem-tip cuttings were harvested from stock plants and inserted immediately in phenolic foam cubes and placed in a glass-glazed greenhouse with a vapor pressure deficit of 0.3 kPa maintained with steam injection on a propagation bench for one week. Overhead mist was provided for 5 s when the integrated light intensity achieved 0.20 mol·m^–2^·h^–1^ or after 60 min, whichever first occurred. The mist contained reverse-osmosis water supplemented with water-soluble fertilizer (MSU Plug Special 13N–2.2P–10.8K; Greencare Fertilizers, Inc.) and micronutrients (M.O.S.T.; JR Peters, Inc., Allentown, PA) providing (mg·L^–1^) 60 nitrogen (N), 10 phosphorous (P), 50 potassium (K), 28 calcium (Ca), 5 magnesium (Mg), 27 sulfur (S), 16 iron (Fe), 10 zinc (Zn), 17 manganese (Mn), 5 copper (Cu), 3 boron (B), and 0.2 molybdenum (Mo). Seeds and seedlings were irrigated overhead daily with reverse osmosis water supplemented with 12N-1.8P-13.4K water-soluble fertilizer. MDT (~23°C) was measured by 0.13-mm type E thermocouples (Omega Engineering). High-pressure sodium lamps provided a photosynthetic photon flux density of ~80 μmol·m^–2^·s^–1^, as measured with a quantum sensor (LI-190R Quantum Sensor; LI-COR Biosciences, Lincoln, NE) every 15 s, and means were logged every hour by a CR-1000 datalogger (Campbell Scientific, Logan, UT) to create a 16-h photoperiod and maintain a target DLI of 10 mol·m^‒2^·d^‒1^.

On 6 Sept. 2017 (rep 1, sweet basil), 20 Oct. 2017 (rep 2, sweet basil), 19 Apr. 2018 (rep 1, purple basil, sage, and spearmint), and 30 Oct. 2018 (rep 2, purple basil, sage, and spearmint), two (spearmint and sweet basil), three (purple basil) or four (sage) weeks after sowing or sticking, the 12 seedlings or rooted cuttings of each crop per treatment were transplanted into hydroponic systems (Active aqua premium high-rise flood table; Hydrofarm) in five connecting glass-glazed greenhouse compartments with target MDTs of constant 23, 26, 29, 32, or 35°C. Each greenhouse contained three hydroponic systems under 0%, ~30%, or ~50% shade cloth (Solaro 3215 D O FB and Solaro 5220 D O; Ludvig Svensson, Kinna, Sweden) used to create DLIs from 5 to 19 mol·m^‒2^·d^‒1^, respectively. Actual temperatures and DLIs are reported in Table 1. The production systems and cultural and environmental control and monitoring equipment used are described by Walters et al. [27], unless reported.

**Table 1:** Average daily light integral (DLI; mol·m^‒2^·d^‒1^ ± sd) and mean daily air temperature (MDT), leaf, and nutrient solution temperatures (°C ± sd) over the three (spearmint and sweet basil), four (purple basil), or five (sage) week growing period for two replications over time. Plants were transplanted on 6 Sept. 2017 (rep 1, sweet basil), 20 Oct. 2017 (rep 2, sweet basil), 19 Apr. 2018 (rep 1, purple basil, sage, and spearmint), and 30 Oct. 2018 (rep 2, purple basil, sage, and spearmint). Data were collected every 15 s with means logged every hour.

	DLI	Temperature°C			DLI	Temperature°C		
Rep.		Air	Leaf	Solution		Air	Leaf	Solution
	Spearmint ‘Spanish’ (*Mentha spicata*)				Purple basil ‘Dark Opal’ (*Ocimum basilicum*)			
1	14.3 ± 2.5	24.3 ± 1.4	-^z^	22.5 ± 1.3	13.7 ± 2.6	24.3 ± 1.6	-	22.7 ± 1.4
	13.7 ± 2.6	24.3 ± 1.4	-	22.3 ± 1.8	13.0 ± 2.6	24.3 ± 1.6	-	22.4 ± 1.8
	9.7 ± 1.7	24.3 ± 1.4	-	22.3 ± 1.5	9.3 ± 1.7	24.3 ± 1.6	-	22.4 ± 1.5
	19.1 ± 3.5	26.6 ± 0.9	27.2 ± 1.5	24.4 ± 0.9	18.6 ± 3.5	26.5 ± 0.9	27.0 ± 1.8	24.4 ± 0.9
	13.0 ± 2.5	26.6 ± 0.9	-	24.2 ± 0.9	12.5 ± 2.6	26.5 ± 0.9	-	24.1 ± 1.0
	8.7 ± 1.8	26.6 ± 0.9	-	24.1 ± 1.0	8.2 ± 1.9	26.5 ± 0.9	-	23.9 ± 1.2
	18.6 ± 3.5	28.9 ± 1.1	28.7 ± 0.9	25.7 ± 0.7	18.0 ± 3.4	28.6 ± 1.1	28.5 ± 1.0	25.8 ± 0.7
	13.4 ± 2.5	28.9 ± 1.1	-	25.2 ± 0.4	12.9 ± 2.6	28.6 ± 1.1	-	25.1 ± 0.4
	9.3 ± 1.7	28.9 ± 1.1	-	25.1 ± 0.5	9.0 ± 1.7	28.6 ± 1.1	-	25.1 ± 0.5
	19.4 ± 3.8	32.5 ± 0.4	-	29.0 ± 1.4	18.7 ± 3.8	32.4 ± 0.4	-	29.4 ± 1.5
	13.6 ± 2.7	32.5 ± 0.4	-	27.3 ± 1.0	13.0 ± 2.7	32.4 ± 0.4	-	27.3 ± 0.9
	9.7 ± 1.7	32.5 ± 0.4	-	26.3 ± 1.2	9.3 ± 1.7	32.4 ± 0.4	-	26.4 ± 1.2
	16.9 ± 3.0	35.1 ± 0.7	35.8 ± 0.8	30.6 ± 0.6	16.2 ± 3.1	35.0 ± 0.7	35.9 ± 0.7	30.6 ± 0.6
	12.2 ± 2.1	35.1 ± 0.7	-	30.0 ± 0.6	11.8 ± 2.1	35.0 ± 0.7	-	30.1 ± 0.6
	9.9 ± 1.6	35.1 ± 0.7	-	29.5 ± 0.7	9.6 ± 1.5	35.0 ± 0.7	-	29.5 ± 0.7
2	10.4 ± 0.9	23.0 ± 1.2	23.1 ± 1.6	22.2 ± 0.9	10.3 ± 1.0	22.9 ± 1.1	22.6 ± 1.6	22.0 ± 0.9
	8.7 ± 0.9	23.0 ± 1.2	-	21.1 ± 0.9	8.6 ± 0.9	22.9 ± 1.1	-	20.8 ± 1.0
	6.5 ± 0.8	23.0 ± 1.2	-	20.8 ± 0.8	6.4 ± 0.8	22.9 ± 1.1	-	20.6 ± 0.8
	10.4 ± 1.3	26.1 ± 0.4	27.0 ± 0.4	23.3 ± 0.6	10.7 ± 1.3	26.1 ± 0.4	27.1 ± 0.5	23.3 ± 0.5
	7.4 ± 0.8	26.1 ± 0.4	-	22.8 ± 0.5	7.3 ± 0.8	26.1 ± 0.4	-	22.8 ± 0.4
	5.0 ± 0.8	26.1 ± 0.4	-	22.6 ± 0.3	5.0 ± 0.7	26.1 ± 0.4	-	22.5 ± 0.3
	9.7 ± 1.0	28.7 ± 0.6	29.8 ± 2.3	25.2 ± 0.4	9.6 ± 1.0	28.7 ± 0.5	29.6 ± 2.0	25.2 ± 0.4
	7.9 ± 0.8	28.7 ± 0.6	-	24.9 ± 0.5	7.9 ± 0.8	28.7 ± 0.5	-	24.8 ± 0.5
	6.8 ± 0.9	28.7 ± 0.6	-	24.2 ± 0.5	6.8 ± 0.9	28.7 ± 0.5	-	24.1 ± 0.5
	11.9 ± 1.2	32.1 ± 1.5	30.1 ± 0.5	27.7 ± 0.3	11.7 ± 1.4	32.1 ± 1.4	30.0 ± 0.5	27.9 ± 0.5
	8.8 ± 0.8	32.1 ± 1.5	-	27.7 ± 1.1	8.7 ± 0.8	32.1 ± 1.4	-	27.5 ± 1.1
	6.1 ± 0.8	32.1 ± 1.5	-	27.7 ± 1.1	6.1 ± 0.7	32.1 ± 1.4	-	27.5 ± 1.1
	11.2 ± 1.0	34.5 ± 0.8	35.4 ± 1.7	30.0 ± 0.8	11.2 ± 1.0	34.4 ± 0.8	34.7 ± 2.0	29.8 ± 0.8
	8.5 ± 0.8	34.5 ± 0.8	-	29.4 ± 0.5	8.4 ± 0.8	34.4 ± 0.8	-	29.5 ± 0.6
	6.6 ± 0.8	34.5 ± 0.8	-	29.1 ± 1.6^y^	6.5 ± 0.8	34.4 ± 0.8	-	28.7 ± 1.6^y^
	Sage ‘Extrakta’ (*Salvia officinalis*)				Sweet basil ‘Nufar’ (*Ocimum basilicum*)			
1	13.3 ± 2.6	24.5 ± 1.5	-	22.9 ± 1.4	12.5 ± 0.8	24.4 ± 1.9	-	24.7 ± 0.9^y^
	12.6 ± 2.8	24.5 ± 1.5	-	22.8 ± 1.8	9.7 ± 0.9	24.4 ± 1.9	-	24.3 ± 1.3
	9.1 ± 1.6	24.5 ± 1.5	-	22.7 ± 1.5	8.1 ± 0.8	24.4 ± 1.9	-	23.6 ± 1.7
	18.3 ± 3.5	26.6 ± 0.9	27.1 ± 1.8	24.6 ± 1.0	13.1 ± 1.7	27.0 ± 0.8	30.1 ± 1.5	26.3 ± 0.8
	12.2 ± 2.7	26.6 ± 0.9	-	24.1 ± 1.0	10.1 ± 1.0	27.0 ± 0.8	-	25.7 ± 0.7
	8.0 ± 2.0	26.6 ± 0.9	-	24.0 ± 1.4	7.7 ± 0.7	27.0 ± 0.8	-	25.7 ± 0.7
	17.6 ± 3.5	28.6 ± 1.1	28.5 ± 1.1	26.0 ± 0.7	13.0 ± 1.7	30.2 ± 1.2	30.7 ± 0.7	28.9 ± 0.9
	12.5 ± 2.6	28.6 ± 1.1	-	25.2 ± 0.4	9.5 ± 1.3	30.2 ± 1.2	-	28.4 ± 1.1
	8.7 ± 1.7	28.6 ± 1.1	-	25.2 ± 0.5	7.2 ± 1.0	30.2 ± 1.2	-	28.0 ± 1.1
	18.3 ± 3.8	32.5 ± 0.4	-	29.8 ± 1.6	11.7 ± 1.6	32.9 ± 0.8	37.0 ± 0.7	30.8 ± 0.4
	12.7 ± 2.7	32.5 ± 0.4	-	27.5 ± 0.9	9.3 ± 1.5	32.9 ± 0.8	-	30.3 ± 0.5
	9.1 ± 1.7	32.5 ± 0.4	-	26.6 ± 1.3	7.0 ± 0.9	32.9 ± 0.8	-	29.3 ± 0.6
	15.8 ± 3.3	35.1 ± 0.6	36.0 ± 0.8	30.8 ± 0.6	12.7 ± 1.1	35.3 ± 1.0	37.4 ± 1.1	32.3 ± 1.0
	11.6 ± 2.2	35.1 ± 0.6	-	30.3 ± 0.7	9.4 ± 0.8	35.3 ± 1.0	-	31.6 ± 0.7
	9.5 ± 1.5	35.1 ± 0.6	-	29.7 ± 0.7	6.3 ± 0.5	35.3 ± 1.0	-	31.9 ± 1.0
2	10.2 ± 1.1	22.8 ± 1.0	22.5 ± 1.5	22.0 ± 0.8	12.9 ± 1.1	23.0 ± 1.3	26.3 ± 1.3	21.7 ± 1.4
	8.5 ± 1.0	22.8 ± 1.0	-	20.8 ± 0.9	10.1 ± 1.0	23.0 ± 1.3	-^z^	21.5 ± 1.1
	6.4 ± 0.8	22.8 ± 1.0	-	20.5 ± 0.8	7.4 ± 0.8	23.0 ± 1.3	-	21.4 ± 1.3
	10.7 ± 1.3	26.1 ± 0.4	27.0 ± 0.5	23.3 ± 0.5	11.1 ± 1.1	25.6 ± 0.6	27.7 ± 0.9	23.9 ± 0.7
	7.2 ± 0.8	26.1 ± 0.4	-	22.8 ± 0.4	9.2 ± 1.0	25.6 ± 0.6	-	23.4 ± 0.7
	4.9 ± 0.7	26.1 ± 0.4	-	22.6 ± 0.3	8.0 ± 0.8	25.6 ± 0.6	-	23.5 ± 0.7
	9.5 ± 1.0	28.7 ± 0.5	29.4 ± 1.9	25.3 ± 0.4	11.3 ± 1.2	28.9 ± 1.9	30.5 ± 0.8	26.0 ± 1.0
	7.8 ± 0.8	28.7 ± 0.5	-	24.9 ± 0.5	8.9 ± 1.1	28.9 ± 1.9	-	25.9 ± 0.9
	6.7 ± 0.9	28.7 ± 0.5	-	24.3 ± 0.5	6.6 ± 0.9	28.9 ± 1.9	-	25.7 ± 1.0
	11.5 ± 1.4	32.0 ± 1.3	29.9 ± 0.5	28.1 ± 0.6	13.2 ± 1.3	31.4 ± 0.7	35.7 ± 1.2	28.7 ± 0.8
	8.5 ± 0.9	32.0 ± 1.3	-	27.5 ± 1.0	9.2 ± 1.0	31.4 ± 0.7	-	27.3 ± 0.8
	6.0 ± 0.7	32.0 ± 1.3	-	27.4 ± 1.0	7.2 ± 0.7	31.4 ± 0.7	-	26.2 ± 0.8
	11.0 ± 1.2	34.5 ± 0.7	34.6 ± 1.8	29.7 ± 0.8	12.0 ± 1.3	35.7 ± 0.8	36.6 ± 0.8	30.3 ± 0.8
	8.3 ± 0.8	34.5 ± 0.7	-	29.7 ± 0.7	8.5 ± 0.9	35.7 ± 0.8	-	29.6 ± 0.7
	6.5 ± 0.8	34.5 ± 0.7	-	28.6 ± 1.4^y^	5.5 ± 0.8	35.7 ± 0.8	-	29.2 ± 0.6

^z^ data not collected.

^y^ partial data reported.

### Growth data collection and analysis

The experiment was organized in a split-plot design with each of five MDTs in separate greenhouse sections and three DLI treatments in each section repeated in time. Plants were harvested when one treatment reached individual marketable size, which was three, four, or five weeks after transplant for spearmint and sweet basil, purple basil, or sage, respectively. The most recent fully expanded leaf of five purple basil, sage, and spearmint in each temperature and DLI treatment were dark acclimated for >15 minutes using manufacturer-supplied clips. Dark-acclimated leaves were exposed to 3,500 μmol·m^–2^·s^–1^ of red radiation (peak wavelength 650 nm) to saturate photosystem II, fluorescence was measured, and maximum quantum yield of dark-adapted leaves (F_v_/F_m_) was calculated and reported by a portable chlorophyll fluorescence meter (Handy Plant Efficiency Analyzer (PEA); Hanstech Instruments Ltd. Norfolk, UK).

The International Commission on Illumination L*a*b color space values of purple basil were measured using a colorimeter (Chroma Meter CR-400; Konica Minolta Sensing; Tokyo, Japan) to quantify foliage coloration. L* is a measure of lightness ranging from 0 (black) to 100 (white), and a* and b* are interpreted on a positive and negative scale. The a* scale ranges from red (positive values) to green (negative values), while b* ranges from yellow (positive values) to blue (negative values). However, these variables are not independent and therefore, cannot be interpreted independently, so Chroma (C*) and hue angle (h°) were calculated [28]. Chroma (C*) is the degree of departure from gray toward pure chromatic color, calculated as $\sqrt{a^{*2}+b^{*2}}$ and representing the hypotenuse of an a*b* plot [28]. Hue angle (h°) is the angle from 0° or 360° on the color wheel. Values 0° and 360° indicate red, 90° is yellow, 120° is green, 180° is bluish green, and 270° is blue. To account for positive and negative a* and b* values, the following equations were used based on McLellan et al. [29]:

If a* and b* are positive: $h{}^{\circ}=\left(\frac{\mathrm{atan}\left(\frac{b^{*}}{a^{*}}\right)}{2\pi }\right)360$

If a* is positive and b* is negative: $h{}^{\circ}=360+\left(\frac{\mathrm{atan}\left(\frac{b^{*}}{a^{*}}\right)}{2\pi }\right)360$

If a* is negative and b* is positive, or if a* and b* are negative: $h{}^{\circ}=180+\left(\frac{\mathrm{atan}\left(\frac{b^{*}}{a^{*}}\right)}{2\pi }\right)360$

Since h° is a continuous circular scale and h° values fell between 0° and 127° or 300° and 360°, values over 300° were transformed by subtracting 360° for data analysis.

The number of branches >2.5 cm and height from the substrate surface to the tip of the tallest leaf were recorded, and leaf area of the four most recent fully expanded leaves was measured with a leaf-area meter (LI-300; LI-COR Biosciences) for 10 plants per treatment. Leaves were separated from stems, and leaf and stem fresh mass of 10 plants per treatment were recorded. Leaf fresh mass fraction was calculated as leaf fresh mass/total fresh mass. Tissue was placed in a forced-air oven maintained at 75°C for at least 3 d, weighed, and dry mass was recorded. Dry matter concentration (DMC) was calculated as g dry mass per kg fresh mass. Analysis of variance was performed using JMP (version 12.0.1, SAS Institute Inc., Cary, NC) and when interactions between replications were not significant, data were pooled. Linear, quadratic, and surface regression analyses were conducted using SigmaPlot (version 11.0, Systat Software Inc., San Jose, CA). Equations used to generate predictive models were based on 300 observations for each species.

## Results

### Purple basil

**Fig 1:**
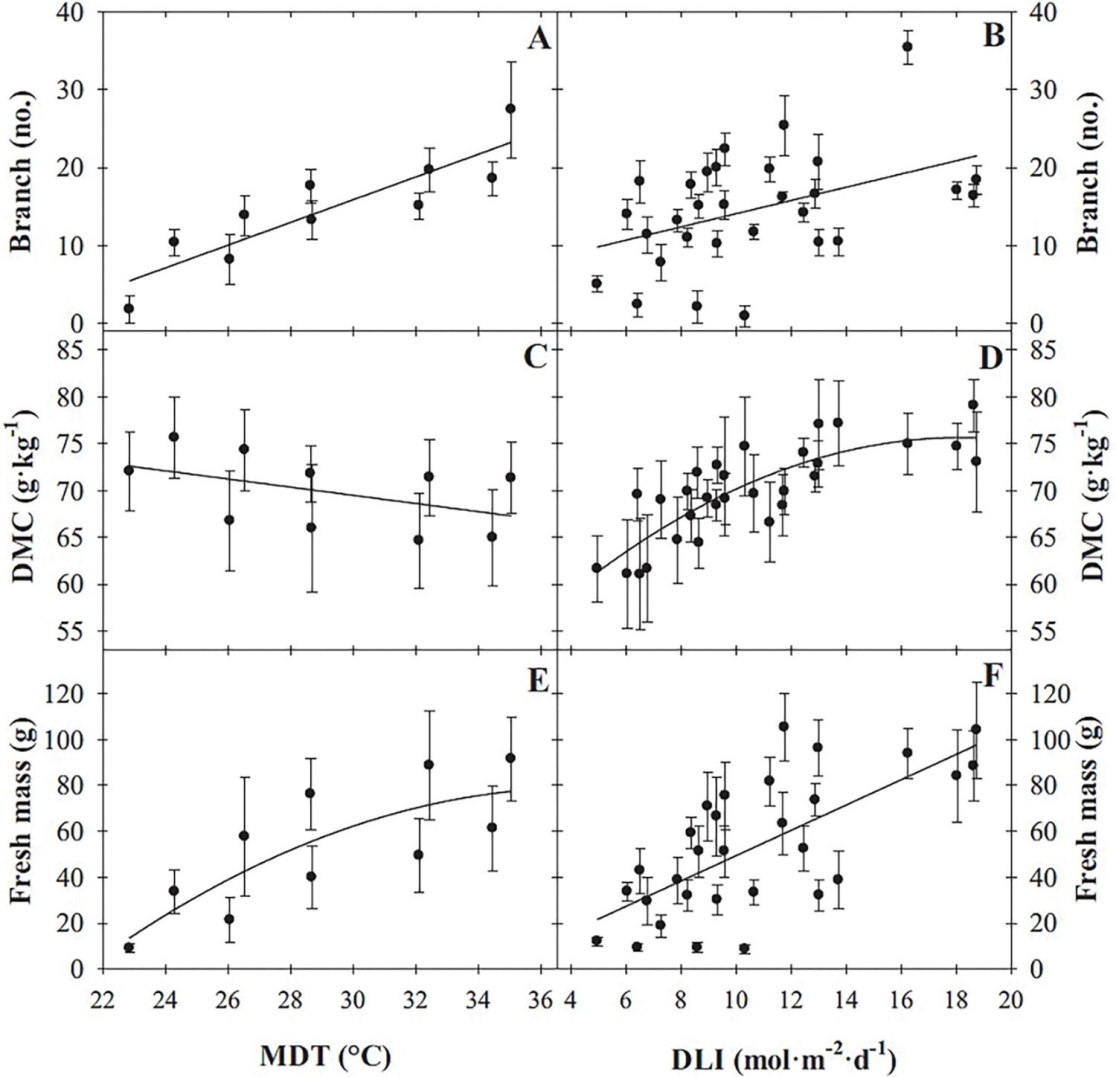
Infleunce of mean daily temperature (MDT) on purple basil ‘Dark Opal’ (*Ocimum basilicum*) branch number (A), dry matter concentration (DMC; C), and fresh mass (E), and infleunce of daily light integral (DLI) on branch number (B), DMC (D), and fresh mass (F). Lines represent model predictions, with the coefficients for these models presented in Table 2. Symbols (means ± sd) represent measured data (A, C, E, n = 27; B, D, F, n = 9).

As the MDT was raised from 22.9 to 35.0°C and DLI increased from 5.0 to 18.7 mol·m^‒2^·d^‒1^, the number of branches purple basil developed increased linearly by 18 and 12 branches, respectively, (Table 2, Fig 1A and 1B). DMC decreased linearly by 8% (5.6 g·kg^‒1^) as MDT increased from 22.9 to 35.0°C and increased quadratically as DLI increased from 5.0 to 18.7 mol·m^‒2^·d^‒1^, with a near-linear increase from 5.0 to 14 mol·m^‒2^·d^‒1^ (61 to 74 g·kg^‒1^) and similar DMCs as DLI further increased to 18.7 mol·m^‒2^·d^‒1^ (76 g·kg^‒1^; Table 2, Fig 1C and 1D). As MDT increased from 22.9 to 35.0°C, fresh mass of purple basil increased quadratically by 4.3-fold (Fig 1E). As DLI increased from 5.0 to 18.7 mol·m^‒2^·d^‒1^, fresh mass increased linearly over 3-fold (by 76 g; Fig 1F).

**Table 2:** Regression analysis parameters and R^2^ or r^2^ for purple basil, sage, spearmint, and sweet basil branch number, height, maximum quantum yield of dark-adapted leaves (F_v_/F_m_), leaf area of four most recently fully expanded leaves, dry matter concentration (DMC), leaf mass fraction, and fresh mass in response to mean daily temperature (MDT;°C) and daily light integral (DLI; mol·m^‒2^·d^‒1^). All models are in the form of: *f* = y0 + a*MDT + b*DLI + c*MDT^2^ + d*DLI^2^ + e*MDT*DLI.

Parameter	y0	(a) MDT	(b) DLI		(c) MDT^2^		(d) DLI^2^	(e) MDT*DLI	R^2^ or r^2^
Purple basil ‘Dark Opal’ (*Ocimum basilicum*)									
Branch (no.)	-2.80E1^z^	1.46		^y^					0.658
	(8.00)^x^	(0.272)							
Branch (no.)	5.58			8.51E-1					0.174
	(3.72)			(3.31E-1)					
Height (cm)	-1.82E2	1.24E1		4.08		-1.94E-1	-9.40E-2	-3.52E-2	0.646
	(1.47E1)	(9.89E-1)		(5.89E-1)		(1.74E-2)	(1.53E-2)	(1.81E-2)	
F_v_/F_m_	4.46E-1	2.11E-2		6.1E-3		-3.E-4		-3.E-4	0.371
	(8.59E-2)	(5.9E-3)		(3.2E-3)		(1.E-4)		(1.E-4)	
Leaf area (cm^2^)	-6.46E2	5.21E1		1.11E1		-9.06E-1		-3.38E-1	0.540
	(7.61E1)	(5.22)		(2.83)		(9.11E-2)		(9.56E-2)	
DMC (g·kg^‒1^)	8.26E1	-4.6E-1							0.087
	(8.61)	(2.93E-1)							
DMC (g·kg^‒1^)	4.85E1			2.99			-8.22E-2		0.419
	(4.92)			(8.72E-1)			(3.57E-2)		
Leaf mass fraction (fresh)	2.75	-1.18E-1		-4.7E-2		1.8E-3	7.E-4	9.E-4	0.615
	(1.30E-1)	(8.7E-3)		(5.2E-3)		(2.E-4)	(1.E-4)	(2.E-4)	
Fresh mass (g)	-3.58E2	2.35E1				-3.15E-1			0.459
	(3.71E2)	(2.58E1)				(4.42E-1)			
Fresh mass (g)	-5.83			5.52					0.420
	(1.23E1)			(1.09)					
Hue angle (h°)	-9.94E2	8.23E1		-3.88E1		-1.47		1.19	0.718
	(9.85E1)	(6.77)		(3.67)		(1.18E-1)		(1.24E-1)	
C*	-3.09E1	1.379							0.601
	(4.33)	(1.47E-1)							
L*	5.33E1	-1.71		-6.99E-1		3.98E-2		1.49E-2	0.687
	(7.98)	(5.47E-1)		(2.97E-1)		(9.6E-3)		(1.00E-2)	
a*	2.32E1	-9.19E-1							0.641
	(2.82)	(9.60E-2)							
b*	-3.07E1	1.32							0.654
	(3.35)	(1.14E-1)							
Sage ‘Extrakta’ (*Salvia officinalis*)									
Branch (no.)	1.92E1	-8.45E-1	1.103		1.45E-2			-2.87E-2	0.376
	(6.90)	(4.73E-1)	(2.65E-1)		(8.0E-3)			(9.0E-3)	
Height (cm)	-2.02E2	1.55E1	4.06		-2.67E-1		-1.31E-1	-4.82E-2	0.367
	(2.40E1)	(1.61)	(9.87E-1)		(2.84E-2)		(2.59E-2)	(3.05E-2)	
F_v_/F_m_	9.04E-1	-2.9E-3							0.251
	(1.99E-2)	(7.E-4)							
Leaf area (cm^2^)	3.42E2	-8.33							0.557
	(2.65E1)	(9.00E-1)							
DMC (g·kg^‒1^)	8.70E1	2.44							0.140
	(4.29E1)	(1.46)							
DMC (g·kg^‒1^)	1.25E2		4.20						0.461
	(1.32E1)		(1.20)						
Leaf mass fraction (fresh)	2.37	-1.05E-1	-3.69E-2		1.6E-3		5.E-4	1.0E-3	0.356
	(1.72E-1)	(1.16E-2)	(7.1E-3)		(2.E-4)		(2.E-4)	(2.E-4)	
Fresh mass (g)	-2.55E2	1.70E1	1.26E1		-2.87E-1		-2.21E-1	-1.93E-1	0.400
	(4.99E1)	(3.35)	(2.05)		(5.91E-2)		(5.68E-2)	(6.35E-2)	
Spearmint ‘Spanish’ (*Mentha spicata*)									
Branch (no.)	-3.79E1	2.83	1.12		-4.25E-2		-2.25E-2	-8.0E-3	0.523
	(6.96)	(4.68E-1)	(2.61E-1)		(8.2E-3)		(6.7E-3)	(8.1E-3)	
Height (cm)	-2.32E2	1.56E1	5.37		-2.46E-1		-9.45E-2	-7.73E-2	0.479
	(2.15E1)	(1.45)	(8.07E-1)		(2.54E-2)		(2.08E-2)	(2.50E-2)	
F_v_/F_m_	5.40E-1	1.66E-2	8.5E-3		-2.E-4			-3.E-4	0.179
	(9.09E-2)	(6.2E-3)	(3.2E-3)		(1.E-4)			(1.E-4)	
Leaf area (cm^2^)	-5.49E2	4.18E1	2.18E1		-7.41E-1		-4.61E-1	-3.07E-1	0.486
	(9.45E1)	(6.35)	(3.54)		(1.11E-1)		(9.12E-2)	(1.10E-1)	
DMC (g·kg^‒1^)	2.69E2	-1.10E1	-3.38		1.71E-1			1.23E-1	0.063
	(5.00E1)	(3.43)	(1.75)		(5.97E-2)			(5.89E-2)	
Leaf mass fraction (fresh)	2.15	-9.27E-2	-3.33E-2		1.5E-3		9.E-4	2.E-4	0.409
	(1.55E-1)	(1.04E-2)	(5.8E-3)		(2.E-4)		(1.E-4)	(2.E-4)	
Fresh mass (g)	-4.18E2	2.52E1	1.33E1		-3.96E-1		-1.86E-1	-1.75E-1	0.601
	(4.91E1)	(3.30)	(1.84)		(5.79E-2)		(4.74E-2)	(5.70E-2)	
Sweet basil ‘Nufar’ (*Ocimum basilicum*)									
Branch (no.)	-1.01E1	1.49E-1	4.46E-2				-7.45E-2	7.01E-2	0.743
	(6.04)	(1.42E-1)	(8.51E-1)				(3.00E-2)	(1.41E-2)	
Node (no.)	-1.54	3.01E-1	2.74E-2		-3.8E-1		-1.42E-2	1.05E-2	0.661
	(2.35)	(1.30E-1)	(2.12E-1)		(2.1E-3)		(7.5E-3)	(3.5E-3)	
Height (cm)	-9.47E1	6.81	1.61		-1.11E-1		-1.17E-1	3.95E-2	0.478
	(1.58E1)	(8.72E-1)	(1.42)		(1.38E-2)		(5.03E-2)	(2.36E-2)	
Leaf area (cm^2^)	-1.05E3	4.89E1	1.76E2		-6.45E-1		-5.09	-2.32	0.485
	(2.91E2)	(1.61E1)	(2.62E1)		(2.55E-1)		(9.28E-1)	(4.36E-1)	
DMC	6.67E1		7.16E-1						0.102
	(2.71)		(2.75E-1)						
Fresh mass (g)	-2.23E2	1.15E1	1.11E1		-1.96E-1		-6.92E-1	2.22E-1	0.632
	(4.35E1)	(2.41)	(3.92)		(3.81E-2)		(1.39E-1)	(6.52E-2)	

^z^ Coefficients for model equations were used to generate Figs 1‒3 and 5‒9.

^y^ Blank cells = 0.

^x^ Standard error (se).

**Fig 2:**
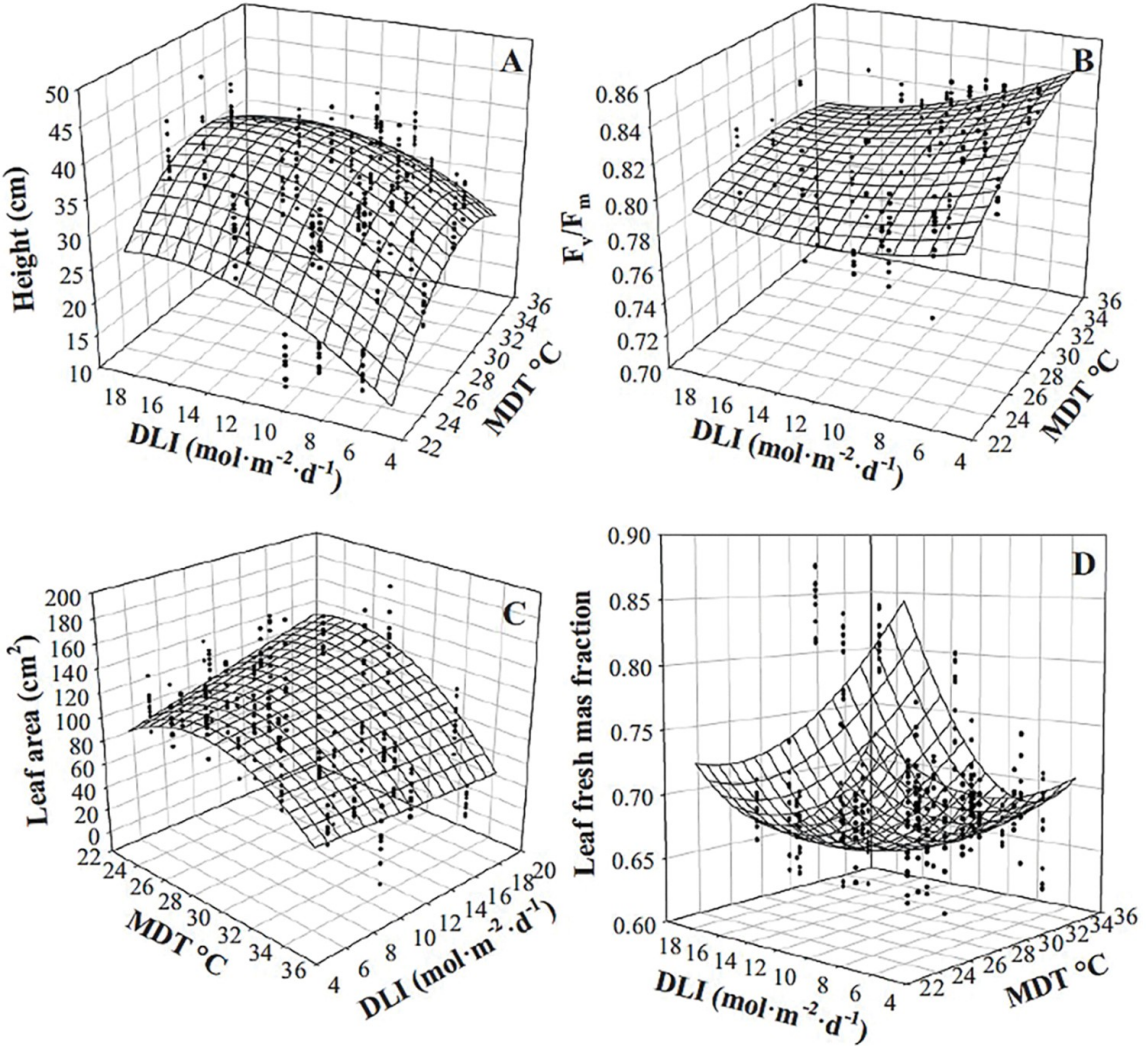
Infleunce of Mean daily temperature (MDT) and daily light integral (DLI) on purple basil ‘Dark Opal’ (*Ocimum basilicum*) height (A), maximum quantum yield of dark-adapted leaves (F_v_/F_m_; B), leaf area (of the four leaves measured; C), and leaf fresh mass fraction (D). Response surfaces represent model predictions. Coefficients for these models are presented in Table 2. Models are each based on 300 individual measurements.

Height of purple basil at harvest increased as MDT increased, but the magnitude was dependent upon DLI and was more pronounced at lower DLIs (Table 2, Fig 2A). Plants were 11.4 and 5.6 cm taller as MDT increased from 22.9 to 35.0°C when the DLI was 5.0 and 18.7 mol·m^‒2^·d^‒1^, respectively. The F_v_/F_m_ was highest (0.80) when the DLI was low (5.0 mol·m^‒2^·d^‒1^) and MDT was high (35.0°C; Table 2, Fig 2B). Reducing MDT or increasing DLI resulted in a reduction of F_v_/F_m_ by up to 0.03 and 0.06, respectively. F_v_/F_m_ values were similar (0.74 to 0.75) regardless of MDT when DLI was high (18.7 mol·m^‒2^·d^‒1^). Smaller leaves developed at higher MDT (Table 2, Fig 2C). For instance, leaf area (of the four leaves measured) was reduced by 81 cm^2^ (60%) as the MDT was raised from 22.9 to 35.0°C under a DLI of 18.7 mol·m^‒2^·d^‒1^. An increase in DLI led to a greater increase in leaf area when the MDT was lower (22.9°C; 46 cm^2^ increase) than when the MDT was higher (35.0°C; 10 cm^2^ increase). MDT and DLI interacted to influence the fraction of total fresh mass comprised of leaves compared to stems (Table 2, Fig 2D). The greatest proportion of leaves occurred when both DLI and MDT were low. At a DLI of 5.0 mol·m^‒2^·d^‒1^ and MDT of 22.9°C, the leaf mass fraction was 88%; regardless of the DLI at an MDT of 35.0°C, the leaf mass fraction was reduced to 77% to 78%.

**Fig 3:**
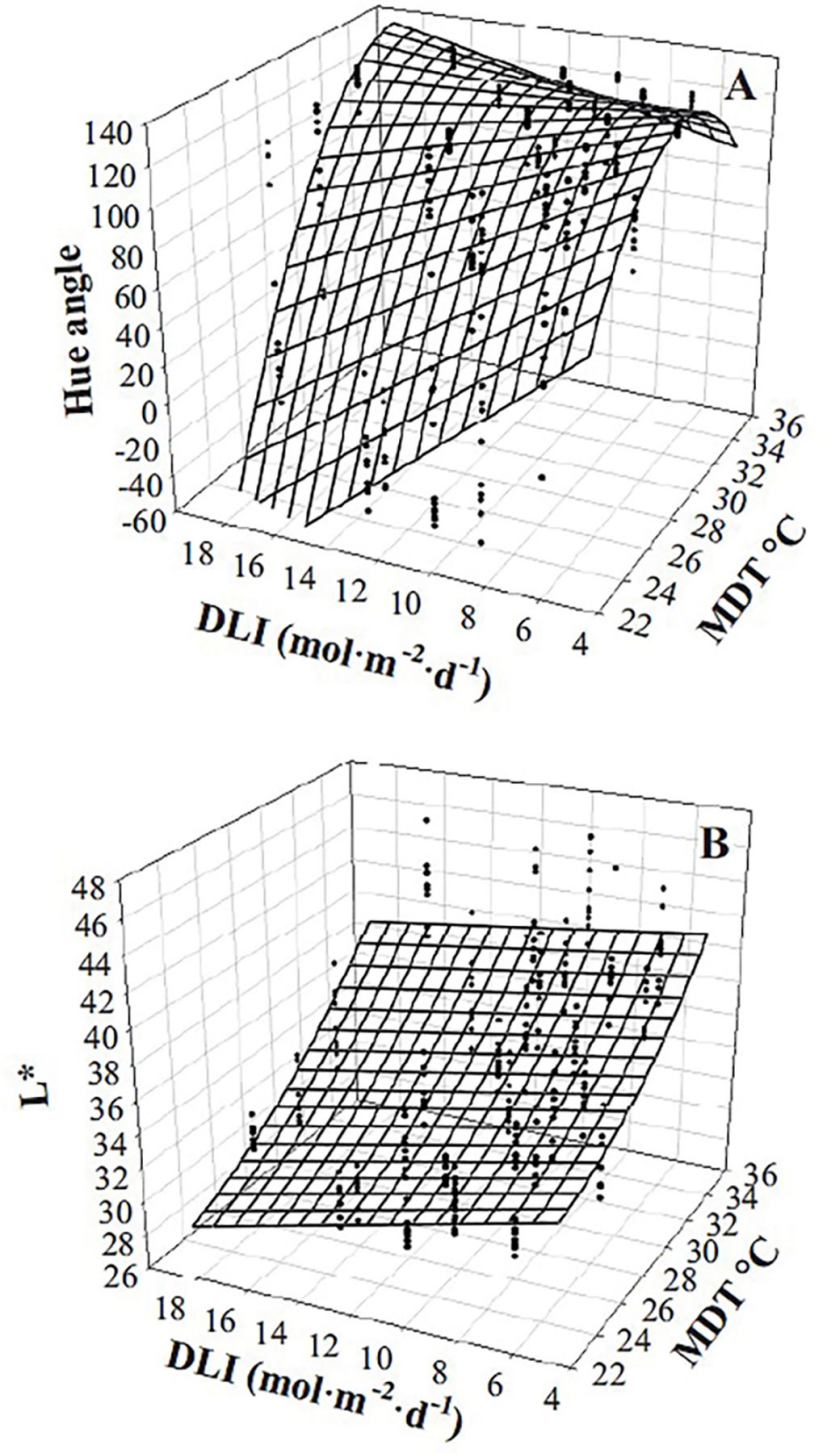
Influence of mean daily temperature (MDT) and daily light integral (DLI) on purple basil ‘Dark Opal’ (*Ocimum basilicum*) hue angle (h°; A) and L* (B). Response surfaces represent model predictions. Coefficients for these models are presented in Table 2. Models are each based on 300 individual measurements.

**Fig 4:**
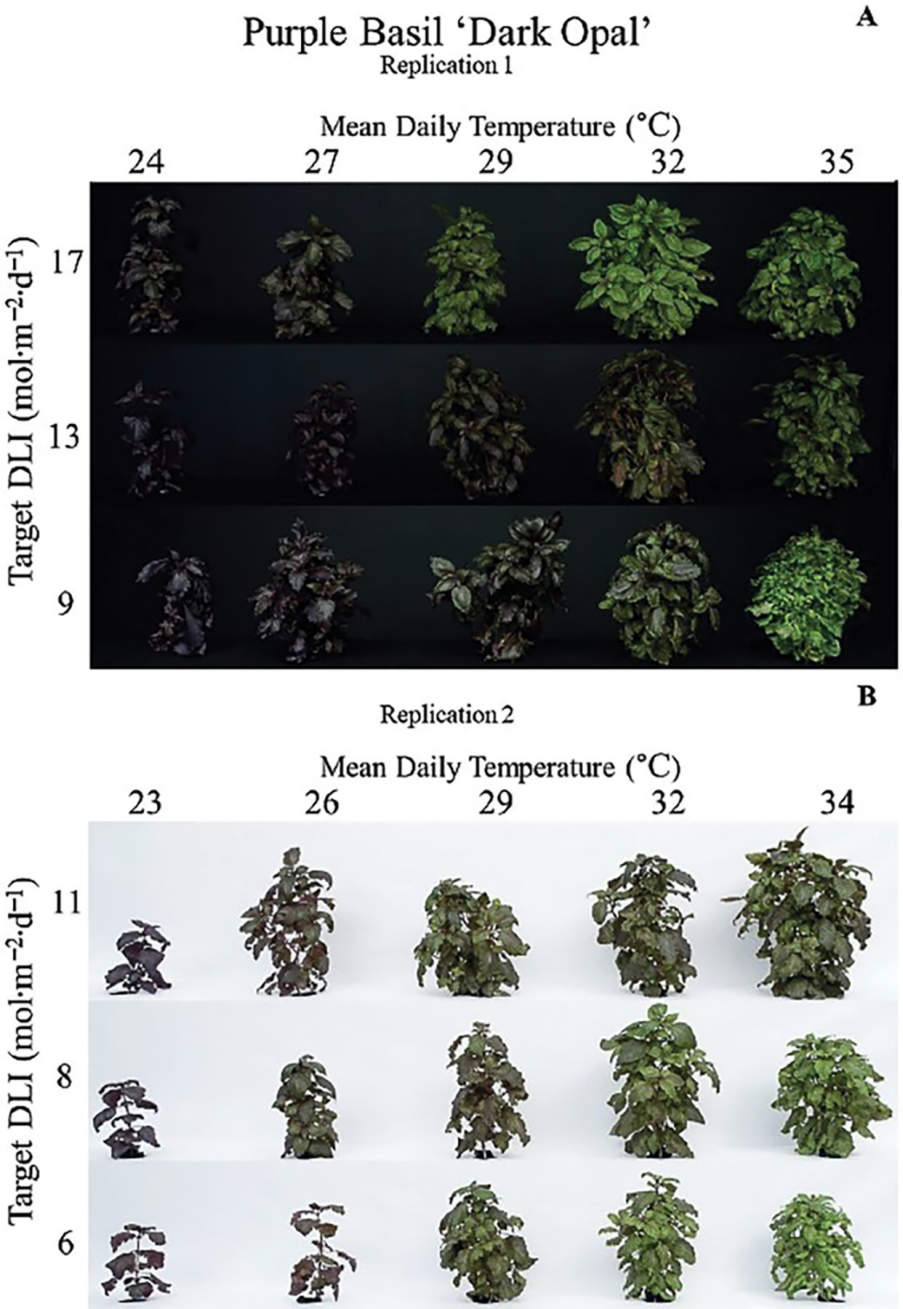
Influence of mean daily temperature (MDT) and daily light integral (DLI) on purple basil ‘Dark Opal’ (*Ocimum basilicum*). Replication 1 (A) was harvested on 17 May 2018, and replication 2 (B) was harvested on 27 Nov. 2018, 7 weeks after sowing and 4 weeks after transplant. Images depict representatives of plants measured.

The interaction of MDT and DLI influenced h° (Table 2, Figs 3A and 4). Purple basil was the reddest/bluest when grown with a high DLI (18.7 mol·m^‒2^·d^‒1^) and low MDT (22.9°C). At an MDT of 24.3°C and DLI of 13.7 mol·m^‒2^·d^‒1^, h° was 333° (-27° calculated). As the MDT increased to 35.0°C, foliage color became greener (138°). The influence of DLI on color was MDT-dependent. At an MDT was 25°C, a reduction in DLI from 18.7 to 5.0 mol·m^‒2^·d^‒1^ led to a h° change from 335° (-25°) to 98° but remained green (99° to 138°) regardless of DLI when MDT was 35.0°C. Purple basil plants were lighter (higher L*) as DLI decreased or MDT increased (Table 2, Fig 3B). L* was influenced by the interaction of MDT and DLI; L* increased more in response to lower DLIs and lower MDTs. For example, as DLI decreased from 18.7 to 5.0 mol·m^‒2^·d^‒1^, L* increased from 28 to 33 at an MDT of 22.9°C and from 39 to 41 at an MDT of 35.0°C. Increasing the MDT resulted in a linear increase in the distance away from greytone; C* increased from 0.7 to 17.3 as MDT increased from 22.9 to 35.0°C (Table 2).

### Sage

**Fig 5:**
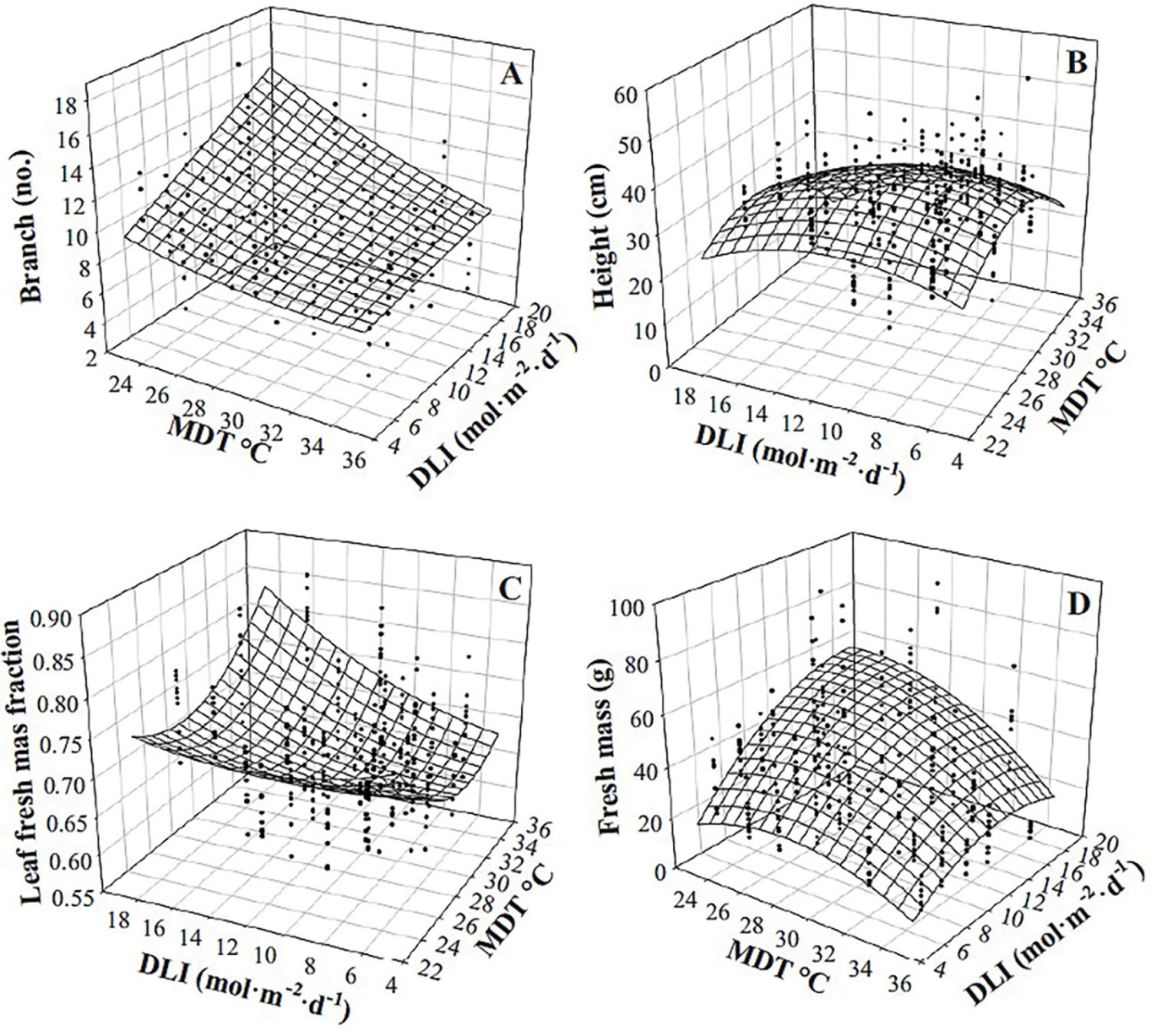
Infleunce of mean daily temperature (MDT) and daily light integral (DLI) on sage ‘Extrakta’ (*Salvia officinalis*) branch number (A), height (B), leaf fresh mass fraction (C), and leaf fresh mass (D). Response surfaces represent model predictions. Coefficients for these models are presented in Table 2. Models are each based on 300 individual measurements.

MDT and DLI interacted to influence the branch number, height, leaf fresh mass fraction, and fresh mass of sage (Table 2, Fig 5A–5D). Branch number increased by 7 (70%) as MDT decreased from 35.1 to 22.8°C, at a DLI of 18.3 mol·m^‒2^·d^‒1^, but branch number only increased by 2 (23%) at a DLI of 4.9 mol·m^‒2^·d^‒1^ (Table 2, Fig 5A). Under a DLI of 18.3 mol·m^‒2^·d^‒1^, plants were 4-cm taller at an MDT of 30.0°C compared to 22.8°C but were 14-cm shorter when grown at 35.1°C than 30.0°C (Fig 5B). Unlike purple basil, the greatest proportion of leaf fresh mass occurred when both DLI and MDT were high (Fig 5C). At a DLI of 18.3 mol·m^‒2^·d^‒1^ and MDT of 35.1°C, the leaf mass fraction was 79% and decreased to 69% at a DLI of 8.1 and MDT of 24.4°C. Fresh mass increased as DLI increased or MDT decreased (Fig 5D). At an MDT of 22.8°C, increasing DLI from 4.9 to 18.3 mol·m^‒2^·d^‒1^ resulted in a 2.4-fold (40.6 g) increase in fresh mass; under the same increase in DLI, fresh mass increased by 90% (8.8 g) at an MDT of 35.1°C. Similarly, when DLI was 4.9 and 18.3 mol·m^‒2^·d^‒1^, reducing the MDT from 35.1 to 22.8°C led to a 2.1-fold (39.1 g) and 75% (7.3 g) increase in fresh mass, respectively.

**Fig 6:**
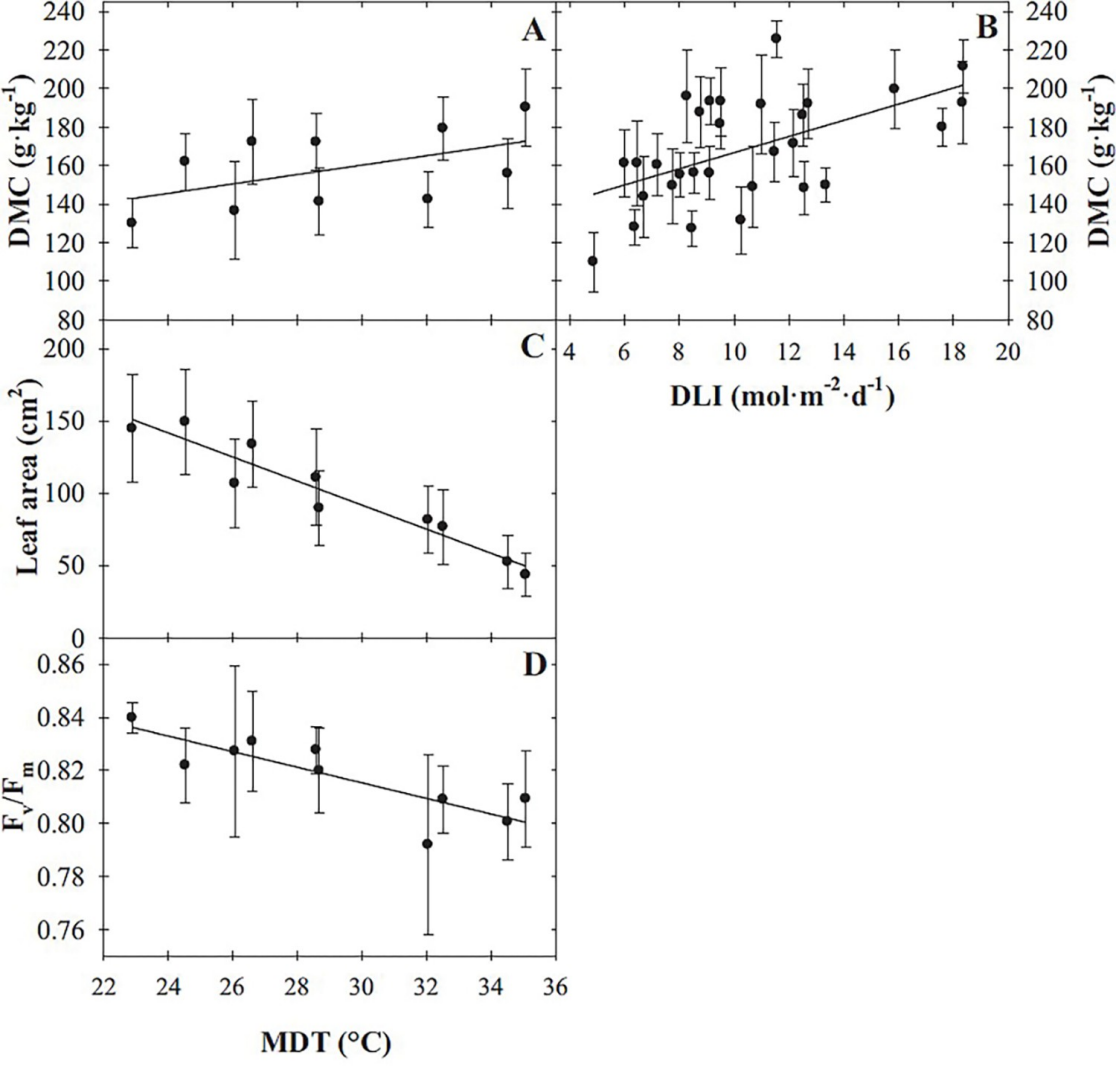
Infleunce of mean daily temperature (MDT) on sage ‘Extrakta’ (*Salvia officinalis*) dry matter concentration (DMC; A), leaf area (C), and maximum quantum yield of dark-adapted leaves (F_v_/F_m_; D), and daily light integral (DLI) effects on DMC (B). Lines represent model predictions, with the coefficients for these models presented in Table 2. Symbols (means ± sd) represent measured data (A, C, D, n = 27; B, n = 9).

Sage DMC increased linearly as both DLI and MDT increased and was not influenced by their interaction (Table 2, Fig 6A and 6B). As MDT and DLI increased from 22.8 to 35.1°C and 4.9 to 18.3 mol·m^‒2^·d^‒1^, respectively, DMC increased by 30 g·kg^‒1^ and 56 g·kg^‒1^. Leaf area (of the four leaves measured) was reduced by 102 cm^2^ (67%) as MDT increased from 22.8 to 35.1°C (Table 2, Fig 6C). Similarly, the same increase in MDT resulted in F_v_/F_m_ decreasing linearly from 0.84 to 0.80 (4%; Table 2, Fig 6D), while DLI did not influence leaf area or F_v_/F_m_.

### Spearmint

**Fig 7:**
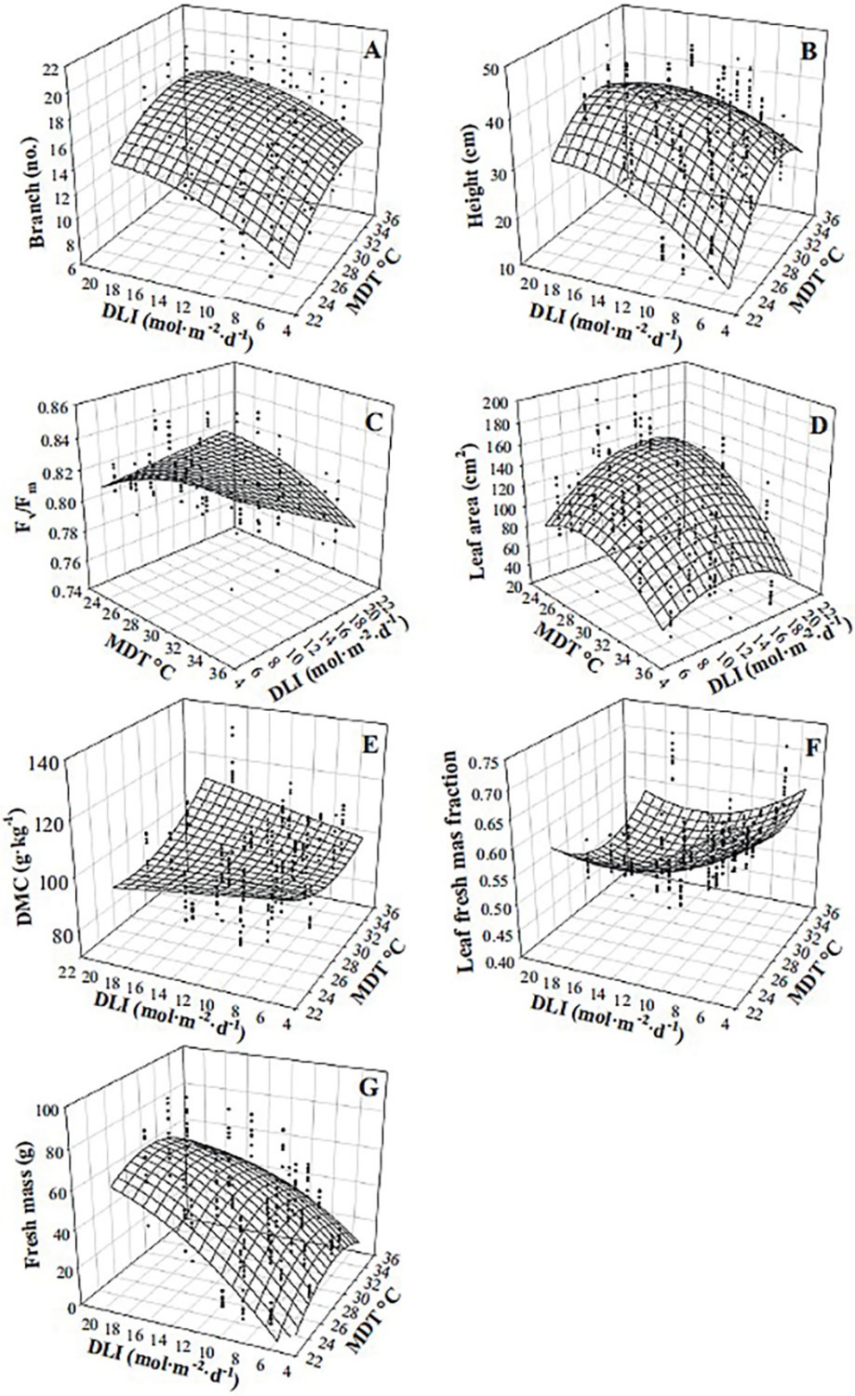
Influence of mean daily temperature (MDT) and daily light integral (DLI) on spearmint ‘Spanish’ (*Mentha spicata*) branch number (A), height (B), maximum quantum yield of dark-adapted leaves (F_v_/F_m_; C), leaf area (of the four leaves measured; D), dry matter concentration (DMC; E), leaf fresh mass fraction (F), and fresh mass (G). Response surfaces represent model predictions. Coefficients for these models are presented in Table 2. Models are each based on 300 individual measurements.

Branch number, height, F_v_/F_m_, leaf area (of the four leaves measured), dry matter concentration, leaf fresh mass fraction, and fresh mass of spearmint were influenced by the interaction of MDT and DLI (Table 2, Fig 7A–7G). Plants were generally taller, had more branches, and produced larger leaves at higher MDTs, but the magnitude of the MDT effect depended upon DLI (Table 2, Fig 7A, 7B and 7D). When DLI was 6.1 mol·m^‒2^·d^‒1^, increasing the MDT from 23.0 to 35.1°C led to 4 more branches (40%), increased height by 15.7 cm (65%), and reduced leaf area by 38 cm^2^ (42%). When DLI was 19.4 mol·m^‒2^·d^‒1^, the same increase in MDT resulted in 2 more branches (17%), and a 2.2 cm (7%) and 88 cm^2^ (67%) reduction in height and leaf area, respectively. F_v_/F_m_ was greatest when the MDT was high (35.1°C) and DLI was low (6.1 mol·m^‒2^·d^‒1^; Table 2, Fig 7C). As the MDT increased from 23.0 to 35.1°C, the DMC increased by 16 g·kg^‒1^ (15%) at a DLI of 19.4 mol·m^‒2^·d^‒1^ (Table 2, Fig 7E). When DLI was 6.1 mol·m^‒2^·d^‒1^, leaf mass fraction decreased from 67% to 62% as MDT increased from 23.0 to 35.1°C, but when DLI was 19.4 mol·m^‒2^·d^‒1^, the same increase in MDT led to a reduction from 59% to 57% (Table 2, Fig 7F). Fresh mass increased by 60 g as DLI increased from 6.1 to 19.4 mol·m^‒2^·d^‒1^ when MDT was 23.0°C and increased by 32 g when MDT was 35.1°C (Table 2, Fig 7G).

### Sweet basil

**Fig 8:**
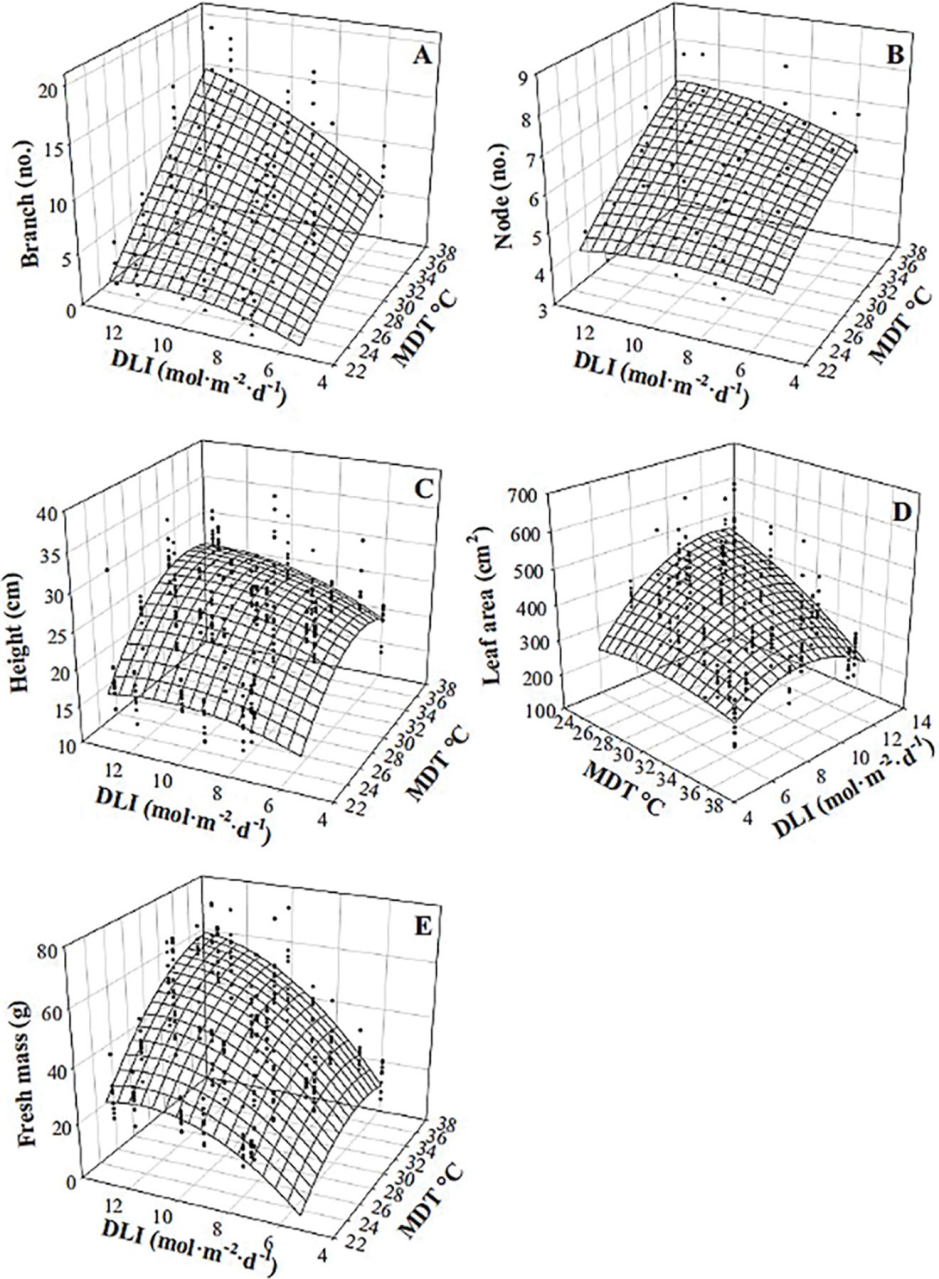
Influence of mean daily temperature (MDT) and daily light integral (DLI) on sweet basil ‘Nufar’ (*Ocimum basilicum*) branch number (A), node number (B), height (C), leaf area (of the four leaves measured; D), and fresh mass (E). Response surfaces represent model predictions. Coefficients for these models are presented in Table 2. Models are each based on 300 individual measurements.

Branch and node number, height, leaf area (of the four leaves measured), and fresh mass of sweet basil responded somewhat similarly to MDT and DLI (Table 2, Fig 8A–8E). Plants were taller, had a larger mass, more branches, and larger leaves as MDT increased, but the magnitude of the MDT effect depended upon DLI. When DLI was 5.5 mol·m^‒2^·d^‒1^, increasing MDT from 23.0 to 35.7°C resulted in the branch number increasing by 7 (from 0), fresh mass by 15 g (3-fold), height by 6.5 cm (48%), and nodes by 2 (39%), and decreased leaf area by 22 cm^2^ (8%). When the DLI was 13.2 mol·m^‒2^·d^‒1^, the same MDT increase led to an increase in branch number by 14 (6.2-fold), fresh mass by 37 g (123%), height by 10.4 cm (65%), and nodes by 3 (62%), and a reduction in leaf area of 248 cm^2^ (53%). F_v_/F_m_ was lowest at 24.4°C (0.77) and was similar (0.81 to 0.83) at higher temperatures (25.6 to 35.7°C; data not shown), while DLI did not influence F_v_/F_m_. Sweet basil DMC increased by 6 g·kg^‒1^ (8%) as DLI increased from 5.5 to 13.2 mol·m^‒2^·d^‒1^ and was not influenced by MDT (Table 2).

## Discussion

### Plant development

Plant development, including leaf unfolding rate and progression towards flowering, are primarily determined by the MDT since it influences the rate of meristematic differentiation. Development can also be influenced by a radiation-induced temperature response (increased meristem temperature) and DLI (increased carbon substrate availability). In the current study, an increase in MDT resulted in an increase in branching for all species grown (Figs 1A, 5A, 7A and 8A). Similarly, Chang et al. [13] found that basil grown at 25 or 30°C developed two more branches than those grown at 15°C. It has also been reported that branches of sweet basil ‘Improved Genovese Compact’ develop faster under higher DLIs [7]. In our study, sweet basil branch number also increased as DLI increased, but the effect was attenuated when MDT was low (23°C; Fig 8A). In contrast, sage and spearmint branch number increased with increasing DLI, but more so at a lower MDT (23°C) compared to 35°C; conversely, purple basil branch number was not influenced by DLI (Figs 5A and 7A). While some developmental responses may be initiated or regulated by radiation, increasing radiation also increases shoot temperature [30]. This results in a radiation-induced temperature response, especially when meristem temperature is impacted, influencing cell differentiation and development.

The height of plants with a central stem is a function of growth and development; both leaf unfolding rate (node number) and internode elongation contribute to height. Many researchers have documented that as DLI and temperature increased, basil height increases [7,13,15,31]. For instance, under 21 d of sole-source lighting, the height of sweet basil ‘Improved Genovese Compact’ increased from 17.4 to 23.3 cm as DLI increased from 9.3 to 16.5 mol·m^‒2^·d^‒1^ [7]. Mortensen [15] reported that as temperature increased from 18 to 27°C, the height of basil increased from 8 to 18 cm. In a separate study, Chang et al. [13] found that basil grown at 15°C were shorter than those grown at 25 or 30°C, but there was no difference in height between basil grown at 25 or 30°C. Lastly, as daytime temperature increased from 18 to 30°C, basil was taller at 90- and 120-days post-germination, but differences were not apparent by 150 d [31]. For sage, plant height increased from 9 to 18 cm as temperature increased from 18 to 27°C [15].

MDT and DLI have been reported to interact and influence height of ornamentals. In campanula ‘Blue Clips’ (*Campanula carpatica*), height at flowering was not influenced by MDT, but by the difference in day and night temperature [32]. The magnitude of difference in plant height to the changing temperature was stronger when the DLI was lower than under medium to high DLIs [32]. In the current study, as MDT increased, the height of purple basil, sage, sweet basil, and spearmint increased to a DLI-dependent maximum height (Figs 2A, 5B, 7B and 8C). For example, height of purple basil increased by 11.4 cm as MDT increased from 22.9 to 35°C when DLI was 5 mol·m^‒2^·d^‒1^, but only increased 5.6 cm when DLI was 18.7 mol·m^‒2^·d^‒1^ (Fig 2A).

A low F_v_/F_m_ is an indicator of plant stress resulting from an inefficiency of photosystem II. Purple basil, spearmint, and sage tended to exhibit lower F_v_/F_m_ values as MDT decreased. Sweet basil had the lowest F_v_/F_m_ at 24.4°C, but similarly higher F_v_/F_m_ among higher MDTs (Figs 2B, 6D and 7C). Similar trends have been demonstrated in sweet basil ‘Nufar’, lemon basil ‘Sweet Dani’ and ‘Lime’, and holy basil, where MDTs from ~11 to ~23°C produced lower F_v_/F_m_ compared to higher MDTs up to 35°C [11]. For purple basil and spearmint, the MDT induced reduction in F_v_/F_m_ was more pronounced when the DLI was lower (Figs 2B and 7C).

### Growth, mass concentration, and partitioning

Our observation of decreased leaf area (of the four leaves measured) with increased MDT across all four crops (Figs 2C, 6D, 7D and 8D) has been documented in many species and cultivars, including spinach ‘Savoy’ (*Spinacia oleracea*) [33], five sunflower (*Helianthus annuus*) cultivars [34], and several tomato (*Solanum lycopersicum*) cultivars [35]. Additionally, DLI can influence leaf area. Dou et al. [7] reported leaf area of sweet basil ‘Improved Genovese Compact’ plants increased from 406 to 614 cm^2^ as DLI increased from 9.3 to 17.8 mol·m^‒2^·d^‒1^. However, the specific leaf area, or area per unit leaf dry weight, decreased from 518 to 398 cm^2^·g^‒1^ as DLI increased. The response of purple basil, spearmint, and sweet basil leaf area to MDT was stronger at higher DLIs, and leaf area tended to increase quadratically as DLI increased (Figs 2C, 7D and 8D). Leaf area of geranium ‘Sooner Red’ (*Pelargonium ×hortorum*) increased as DLI increased from <5 to 20 mol·m^‒2^·d^‒1^ [36] but decreased in geranium ‘Red Elite’ and heliconia ‘Golden Torch’ (*Heliconia psittacorum ×spathocircinata*) as DLI increased from ~20 to ~30 mol·m^‒2^·d^‒1^ [6,37,38].

Dry matter concentration is an indication of plant quality; higher DMC is desired and is an indication of the commercially used term “toning” [39]. In general, DMC increased as DLI increased in our study, but the influence of MDT on DMC was crop dependent. As MDT increased, purple basil DMC decreased, while sage and spearmint DMC increased (Figs 1C, 6A and 7E). Similar to sage and spearmint, Dou et al. [7] reported DMC of sweet basil ‘Improved Genovese Compact’ increased linearly from 67 to 92 g·kg^‒1^ as DLI increased from 9.3 to 17.8 mol·m^‒2^·d^‒1^. In another study, water content of mint, sage, and sweet basil decreased by 5.2%, 6.1%, and 2.7% as DLI increased from 2 to 20 mol·m^‒2^·d^‒1^ [40], and DMC of ornamentals including ageratum ‘Hawaii White’ (*Ageratum houstonianum*), begonia ‘Vodka Cocktail’ (*Begonia ×semperflorens-cultorum*), impatiens ‘Cajun Red’, marigold ‘American Antigua Orange’, petunia ‘Apple Blossom’, salvia ‘Lady in Red’ (*Salvia coccinea*), vinca ‘Pacific Lilac’ (*Catharanthus roseus*), and zinnia ‘Dreamland Rose’ (*Zinnia elegans*) increased linearly or quadratically as DLI increased from 4.8 to 42.9 mol·m^‒2^·d^‒1^ [39].

In a meta-analysis of biomass allocation patterns to leaves, stems, and roots, Poorter et al. [41] determined that the fraction of whole-plant mass represented by leaves most strongly decreased as DLI increased up to ~20 mol·m^‒2^·d^‒1^, above which the response saturated. Additionally, increasing temperature increased leaf mass fraction, though the response was attenuated compared to increasing DLI [41]. In our study, MDT and DLI interacted to influence leaf mass fraction. Congruent with the meta-analysis, increasing sage and spearmint DLI and sage MDT increased leaf mass fraction quadratically (Figs 5C and 7F). In contrast, increasing the DLI for purple basil and MDT for spearmint and purple basil led to a quadratic decrease in leaf mass fraction (Figs 2D and 7F).

### Fresh mass modeling

A temperature response curve can be utilized to calculate fresh mass in response to MDT. For example, as MDT increased from 18 to 35°C, dry mass MDT_opt_ of six sage species (*Salvia* spp.) occurred at MDTs ranging from 21 to 35°C but varied among species [14]. For common sage, fresh mass increased from 37 to 72 g as temperature increased from 18 to 27°C [15]. The influence of MDT on sweet basil fresh mass has been studied extensively [11,13,31,42]. The T_b_ has been reported to range from 10.9 to 12.1°C, and the T_opt_ has been found to range from 25 to 35°C [11,13,31,42]. Fresh mass of purple basil in our study increased quadratically as MDT increased from 22.9 to 35.0°C (Fig 1E). Although supraoptimal MDTs were not observed and the calculated MDT_opt_ is beyond the experimental range, using the regression equation, the extrapolated MDT_opt_ is 37.2°C; however, this prediction may not be accurate. While similar trends in sweet basil were observed, in that fresh mass increased as MDT increased from 23 to ~35°C, we determined that MDT and DLI interacted not only to influence sweet basil, but also sage and spearmint fresh mass (Figs 5D, 7G and 8E).

In general, fresh mass of purple basil, sage, spearmint, and sweet basil increased either linearly or quadratically as DLI increased (Figs 1F, 5D, 7G and 8E). Purple basil fresh mass increased linearly from 21.8 to 97.4 g as DLI increased from 5.0 to 18.7 mol·m^‒2^·d^‒1^. Other researchers observed similar trends; as DLI increased from 9.3 to 17.8 mol·m^‒2^·d^‒1^, sweet basil ‘Improved Genovese Compact’ fresh mass increased linearly from 13.1 to 23.3 g [7] and increasing DLI from 2 to 20 mol·m^‒2^·d^‒1^ increased sweet basil ‘Nufar’ fresh mass linearly by 136.4 g [40]. Mint and sage fresh mass increased as DLI increased from 2 mol·m^‒2^·d^‒1^ to an optimum DLI (DLI_opt_) of 14.9 and 15.9 mol·m^‒2^·d^‒1^, respectively [40].

**Fig 9:**
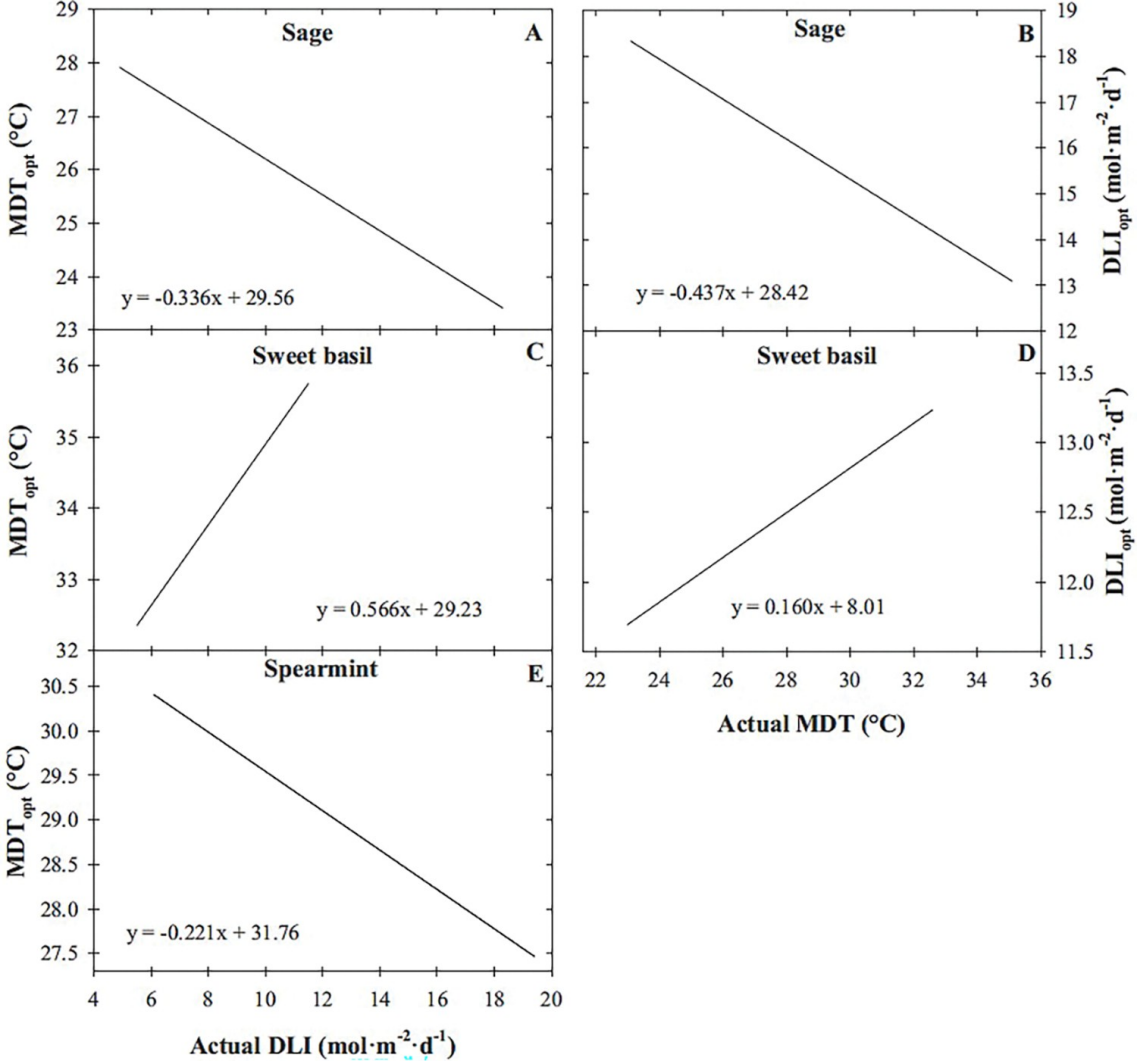
Predicted optimal mean daily temperature (MDT_opt_) to achieve the greatest sage ‘Extrakta’ (*Salvia officinalis*; A), sweet basil ‘Nufar’ (*Ocimum basilicum*; C), and spearmint ‘Spanish’ (*Mentha spicata*; E) fresh mass based on actual daily light integral (DLI). Also, predicted DLI_opt_ to achieve the greatest sage (B) and sweet basil (D) fresh mass based on actual MDT. Equations were generated based on a surface regression model (Figs 5D, 7G and 8E) with model coefficients reported in Table 2.

Since MDT and DLI interacted to influence sage, spearmint, and sweet basil fresh mass, determining MDT_opt_ or DLI_opt_ based on other environmental factors can improve production efficiency to achieve desired growth and energy-use efficiency outcomes, especially since the optimal MDT or DLI may shift as environmental factors interact. Additionally, the ability to calculate MDT_opt_ and DLI_opt_ requires supraoptimal temperatures. In this study, we chose fresh mass as the parameter to calculate MDT_opt_ and DLI_opt_ for, but supraoptimal temperatures were achieved for many parameters measured in this study and MDT_opt_ and DLI_opt_ could be calculated for those as well. Therefore, similar to the methods used in [27], surface regression models (Table 2, Figs 5D, 7G and 8E) were used to calculate the MDT_opt_ for each parameter based on DLI by using *f*_*M*DTopt_ = *a* + 2*c*(*MDT*) + *b*(*DLI*) = 0, and the DLI_opt_ based on MDT using *f*_*DLI*opt_ = *b* + 2*d*(*DLI*) + *e*(*MDT*) = 0 (Fig 9A–9E). For example, as DLI increased from 6 to 18 mol·m^‒2^·d^‒1^, fresh mass of sage and spearmint MDT_opt_ decreased from 27.5 to 23.5°C and from 30.4 to 27.8°C, respectively (Fig 9A and 9E). In contrast, as DLI increased from 6 to 11 mol·m^‒2^·d^‒1^, fresh mass of sweet basil MDT_opt_ increased from 32.6 to 35.5°C (Fig 9C). Similarly, as MDT increased from 24 to 32°C, the fresh mass DLI_opt_ of sage decreased from 17.9 to 14.4 mol·m^‒2^·d^‒1^, while sweet basil fresh mass DLI_opt_ increased from 11.8 to 13.2 mol·m^‒2^·d^‒1^ (Fig 9B and 9D). The DLI_opt_ for spearmint and MDT_opt_ for purple basil could not be calculated because supraoptimal DLIs and MDTs, respectively, were not achieved.

### Color

For purple basil, foliage color is an additional quality parameter, alongside plant mass, contributing not only to visual appeal but also serving as an indicator of anthocyanin concentration [43]. Anthocyanins are desired in food crops due to their antioxidant activity, potentially increasing the nutritional value when consumed, and their contribution to the red and purple color of crops.

Anthocyanins function largely as defense molecules. For example, increased anthocyanin concentration can impart freezing tolerance or protect against excess radiant energy [44]. As described previously, temperature plays a large role in the kinetics of plant enzymatic reaction rates. In general, as temperature increases above T_b_, enzymatic activity increases. Beyond an enzymatic-dependent T_max_, enzymes begin to denature and cease in their functions. However, enzymes can also be up- or down-regulated by increases or decreases in temperature. One major enzyme in the anthocyanin biosynthesis pathway, phenylalanine ammonia lyase, is upregulated by decreasing temperatures [45]. As temperatures decrease, anthocyanin biosynthesis and accumulation increases [46]. This is mainly due to reduced anthocyanin degradation [19] and reduced high-temperature dependent degradation of ELONGATED HYPOCOTYL 5 protein, a positive regulator of anthocyanin biosynthesis at multiple points in the pathway [46]. In crops such as grape (*Vitis vinifera*) and apple (*Malus domestica*), researchers have reported reduced anthocyanin concentrations as temperatures increase [16–19]. In the current study, increasing purple basil MDT (regardless of DLI) increased the green coloration quantified by a ~120 h°, low a*, and high b* (Table 2, Figs 3A and 4). The greener plants were also lighter (L*) colored (Fig 3B).

Anthocyanins can also accumulate in response to increased DLI or radiation intensities, functioning as photoprotectants [44]. When MDT is low, increasing DLI is an effective strategy to increase the red/blue (purple) color quantified by a ~260 h°, increased a*, and reduced b* (Fig 3A). In addition to increasing the greenness of purple basil, increasing MDT also increased the C* or magnitude of colorfulness (Table 2).

If temperature is the primary environmental tool a grower can manipulate to enhance fresh mass yield, product quality should be taken into account. If yield is the main production factor and purple coloration is secondary, green sweet basil cultivars could be grown instead of purple basil cultivars due to their much higher growth rate [47].

## Conclusions

These data and models generated allow us to predict plant growth, development, and leaf coloration in response to MDT and DLI, and to conduct energy-benefit analyses. Though technology to manipulate the growing environment exists, these data will allow growers to calculate the most advantageous growing environment (taking growth, development, quality, and energy into account) and utilize their technologies to realize it. In addition to aiding growers in manipulating the environment for specific culinary herb crops, comparisons can be made to group or separate plants based on growth rates and favorable production environments. Different cultivars may respond differently to MDT and DLI. These models can serve as a basis for environmental controls with general trends informing production decisions and estimating increased (or decreased) growth, development, and quality potential. Additionally, these data can contribute to future multi-parameter machine-learning models.
